# Association of constitutively activated hepatocyte growth factor receptor (Met) with resistance to a dual EGFR/Her2 inhibitor in non-small-cell lung cancer cells

**DOI:** 10.1038/sj.bjc.6604937

**Published:** 2009-02-24

**Authors:** S Agarwal, C Zerillo, J Kolmakova, J G Christensen, L N Harris, D L Rimm, M P DiGiovanna, D F Stern

**Affiliations:** 1 Department of Pathology, Yale University School of Medicine, New Haven, CT 06520, USA; 2 Department of Internal Medicine, Section of Medical Oncology, Yale Comprehensive Cancer Center, Yale University School of Medicine, New Haven, CT 06520, USA; 3 Department of Research Pharmacology, Pfizer Global Research and Development, La Jolla, CA 92121, USA

**Keywords:** Met, Her/ErbB receptor family, lung cancer, signal transduction, RTK

## Abstract

There is a pressing need to identify new drug targets and novel approaches for treatment of non-small-cell lung carcinoma (NSCLC). Members of the epidermal growth factor receptor (EGFR) and Met receptor families have been identified as important molecular targets for NSCLC. Two EGFR tyrosine kinase inhibitors (TKIs; erlotinib and gefitinib) are in current clinical use, but a majority of patients do not respond to these targeted therapies. We used receptor TK (RTK) capture arrays to identify receptors active in NSCLC cell lines. As Met and ErbBs were active, we explored the potential therapeutic advantage of combined targeting of Met with ErbB receptor family inhibitors for treatment of NSCLC. We found that Met physically interacts with both EGFR and Her2 in a NSCLC cell line with overexpression/overactivation of Met. Combined use of a dual EGFR/Her2 inhibitor with a Met inhibitor yields maximal growth inhibition compared with the use of EGFR and/or Met inhibitors. This suggests that simultaneous inhibition of multiple RTKs may be needed to effectively abrogate tumour cell growth. Phosphoproteomic analysis by RTK capture arrays may be a valuable tool for identifying the subset of tumours with functional receptor activation, regardless of mechanism.

Non-small-cell lung cancer (NSCLC) accounts for the majority of all lung cancer cases and, with poor prognosis and low cure rate, is a leading cause of cancer mortality. Tyrosine kinase inhibitors (TKIs) have emerged as an important class of drugs for the treatment of NSCLC. Gefitinib (Iressa) and erlotinib (Tarceva) are the two US FDA-approved epidermal growth factor receptor (EGFR)-specific TKIs that have shown clinical benefit in treating NSCLC. They appear to work, in part, by inhibiting the effects of gain-of-function mutations in EGFR, but patients with tumours lacking mutations benefit as well (Sordella *et al*, 2004; Mukohara *et al*, 2005). Gain-of-function mutations are clustered around the catalytic domain of EGFR and are either single amino-acid substitutions or small insertions/deletions. However, not all patients carrying activating mutant EGFR benefit from TKI therapy and almost all acquire resistance within a year after initial response (Haber *et al*, 2005; Riely *et al*, 2006; Sharma *et al*, 2007).

A major mechanism of resistance to TKI-based therapy is the development of a second site mutation (T790M) in EGFR causing a conformational change that inhibits effective binding of the kinase inhibitors to the ATP pocket (Liu *et al*, 2006). Another secondary mutation (D761Y) in EGFR was identified in an NSCLC brain metastasis originating from a primary tumour that initially responded to gefitinib-based therapy (Balak *et al*, 2006). Another route for escape from TKI therapy is the acquired amplification of the hepatocyte growth factor (HGF) receptor, *MET*, which was found in four of eighteen (22%) resistant NSCLC (Engelman *et al*, 2007). The majority of the amplifications were found in metastatic lesions, suggesting that Met may be involved in the development of metastases as well as acquired resistance to TKI therapy.

Met plays a critical role in cancer, liver and kidney regeneration and mammary gland development, including cell proliferation, motility, invasion and branching tubulogenesis (Yang *et al*, 1995; Rosario and Birchmeier, 2003; Gao and Vande Woude, 2005; Gao *et al*, 2005; Desiderio, 2007; Sattler and Salgia, 2007). Met is present in all tissue types, and activation of Met by its ligand, HGF, leads to the activation of several signalling pathways that coordinate to achieve Met-dependent cellular functions. In normal cells, Met localises predominantly at the plasma membrane; however, in the germinal tissue layer of normal colon, skin and testis, and in cancerous tissue, both cytoplasmic and nuclear localisation have been observed (Pozner-Moulis *et al*, 2006). Gain-of-function mutations, overexpression or amplification of *MET* have been identified and are associated with tumour growth and metastasis (Ma *et al*, 2003; Lengyel *et al*, 2005; Kong-Beltran *et al*, 2006).

Although a small fraction of NSCLC patients (∼10%) have major objective responses to EGFR-based therapy, the majority of NSCLC patients do not respond to EGFR-targeted therapies. Thus, there is a pressing clinical need for the identification of new drug targets and new treatment strategies. It is known that EGFR signalling is modulated by other receptor tyrosine kinases (RTKs). For example, it is well established that heterodimerisation with other ErbB family receptors, Her2 and Her3, augments the oncogenic activities of EGFR (Engelman *et al*, 2005, 2007; Arteaga, 2007). Furthermore, recent evidence implicates Met in functional interactions with EGFR and Her3 (Jo *et al*, 2000). As both the ErbB family of receptors and Met are promising molecular targets for therapy of NSCLC, and with evidence for functional interactions of these receptors, we have explored the possibility that combined targeting of Met and one or more ErbB family members may have therapeutic promise.

## Materials and methods

### Cell lines and other reagents

H441 and H1666 cells were purchased from ATCC (Manassas, VA, USA) and were maintained in RPMI supplemented with 10% FBS, sodium pyruvate, glutamine, penicillin and streptomycin in a 37°C incubator containing 5% CO_2_. 32D/Met cells were generously provided to us by Dr Donald Bottaro from the National Cancer Institute, Bethesda, MD, USA. These cells were maintained in RPMI medium with 10% WEHI-conditioned medium to provide IL-3 (Day *et al*, 1999). PHA665752 (a small molecule TKI for Met) was a generous gift from Pfizer (La Jolla, CA, USA), GW2974 (a dual small molecule TKI for both EGFR and Her2) was purchased from Calbiochem (Gibbstown, NJ, USA) and gefitinib (a small molecule TKI for EGFR) was purchased from Biaffin GmbH & Co KG (Kassel, Germany). All drugs were dissolved in DMSO to produce 20-mM stock solutions. Rabbit anti-EGFR, mouse anti-EGFR, rabbit anti-Met, rabbit anti-Her2, mouse anti-Her3, mouse IgG, goat antimouse HRP and goat antirabbit HRP antibodies were purchased from Santa Cruz Biotechnology (Santa Cruz, CA, USA); mouse anti-Her2 was purchased from Labvision (Fremont, CA, USA); rabbit anti-Her3, rabbit anti-Akt, rabbit anti-phospho-Akt, rabbit anti-Erk1/2, rabbit anti-phospho-Erk1/2, mouse antiphosphotyrosine, mouse anti-Stat3, rabbit antiphospho-Stat3 (Ser 727), rabbit antiphospho-Stat3 (Y705), mouse anti-Met, rabbit antiphospho-Met (Y1234/1235), rabbit antiphospho-EGFR (Y1068), rabbit antiphospho-EGFR (Y992), rabbit antiphospho-EGFR (845) and rabbit anti-*β*-tubulin were purchased from Cell Signalling Technology (Danvers, MA, USA); rabbit anti-Shc was purchased from Upstate Cell Signalling Solutions (Billerica, MA, USA); and rabbit antiphospho-Shc was purchased from Sigma-Aldrich (St Louis, MO, USA). Epidermal growth factor (EGF), HGF and human phospho-RTK array kits were purchased from R&D Systems (Minneapolis, MN, USA).

### Receptor tyrosine kinase antibody array profile

**Figure 1:**
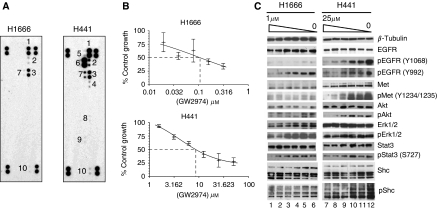
Activation of Met and response to GW2974 in H441 cells. (**A**) Multiple RTKs are activated in H441 and H1666 cells in full serum conditions. Whole cell extracts (200 *μ*g) were incubated with RTK capture array membranes. RTK activation was determined by probing with phosphotyrosine antibody conjugated to horse-radish peroxidase. Paired spots correspond to 1: EGFR; 2: Her2; 3: Her3; 4: Her4; 5: Mer; 6: Met; 7: MSPR; 8: Flt3; 9: Ret; and 10: Dtk. The four sets of duplicate spots at each corner of the RTK array membrane serve to orient and align the membrane to identify and correlate the positive set of spots to individual RTKs. (**B**) Sensitivity of cell lines to GW2974. Cells were treated with indicated concentrations of GW2974 for 5 days, and cell proliferation was measured using a WST-1 colorimetric assay. The GI_50_ of H1666 is 0.1 *μ*M compared with 8.6 *μ*M for H441 cells. Error bars represent s.d. of three data sets. Representative data are shown from multiple experiments. (**C**) Dose-dependent inhibition of EGFR-dependent signalling in H1666 cells and Met-dependent signalling in H441 cells by GW2974. Whole cell extracts were made from cells treated with GW2974 at indicated concentrations for 5 days as in panel B, and subjected to immunoblotting with the indicated antibodies. Arrows indicate respective protein bands. *β*-Tubulin was used as a loading control. GW2974 concentration in lanes 1: 1 *μ*M; 2: 0.5 *μ*M; 3: 0.25 *μ*M; 4: 0.125 *μ*M; 5: 0.0625 *μ*M; 6: 0 *μ*M; 7: 25 *μ*M; 8: 12.5 *μ*M; 9: 6.25 *μ*M; 10: 3.125 *μ*M; 11: 1.56 *μ*M and 12: 0 *μ*M.

**Figure 2:**
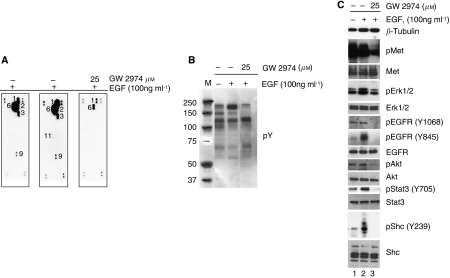
GW2974 inhibits constitutively activated Met, Her2 and Her3 in H441 cells. (**A**) GW2974 abrogates the activation of multiple receptors in H441 cells. Cells were starved for 24 h and then treated for 2 h with GW2974 before incubation with EGF (100 ng ml^−1^) for 10 min. Whole cell extracts (500 *μ*g) were analysed on each RTK array membrane, and activation status of receptors was assessed using antiphosphotyrosine antibody and numbered as in Figure 1A. 1: EGFR; 2: Her2; 3: Her3; 6: Met; 9: Ret; and 11: VEGFR-2. (**B** and **C**) GW2974 inhibits Met signalling and EGF-activated signalling. The extracts analysed in panel A were analysed by immunoblotting with indicated antibodies. M and numbers in panel B indicate molecular weight marker for proteins. *β*-Tubulin was used as a loading control.

Either 200 *μ*g (Figures 1A and 5A) or 500 *μ*g (Figure 2A) of whole cell extracts were analysed on human phospho-RTK arrays from R&D Systems according to the manufacturer's recommendation. Details of the protocol are provided in the Supplementary section.

### Immunoprecipitation (IP) and immunoblot

For IP experiments, cells were incubated with either vehicle (0.5% DMSO) or with indicated concentrations of GW2974 in 0.5% DMSO containing cell media for 2 h. Detailed protocols are in the Supplementary section.

### Cell proliferation assay

Cells were plated on 96-well plates in 50 *μ*l of media at a density of 3000 cells per well for H441 cells or 10 000 cells per well for H1666 cells. Compounds were serially diluted into cell media as 2 × stocks containing 1% DMSO, and 50 *μ*l of each dilution was added to the wells in triplicate. After 5 days of incubation with the compounds, cell survival was assessed by adding 10 *μ*l of WST-1 [2-(4-iodophenyl)-3-(4-nitrophenyl)-5-(2,4-disulphophenyl)-2H tetrazolium] reagent (Roche Applied Sciences, Indianapolis, IN, USA) in each well of a 96-well plate. After incubation for 3 h, the plates were read in a plate reader (Spectramax 250; Molecular Devices, Sunnyvale, CA, USA) at 450 nm. The zero time for cells with only 0.5% DMSO was also assessed for each plate and was subtracted from the 5-day time points. The XLfit program (ID Business Solutions, Parsippany, NJ, USA) was used to obtain and calculate the GI_50_ curves for each compound using a non-linear regression curve fit utilising Lavenburg–Marquardt algorithm. Each experiment was performed a minimum of three times.

### Hepatocyte growth factor activation

Cells were plated in 24-well plate in 750 *μ*l of starvation media (no FBS and no IL-3 containing WEHI media) at a density of 5 × 10^6^ cells per well for 32D/Met cells. After 2 h of starvation, compounds were serially diluted into cell starvation media as 2 × stocks containing 1% DMSO, and 750 *μ*l of each dilution was added to the wells. Details of the protocol are in the Supplementary section.

### Lentivirus infection for Met shRNA

Met shRNA cloned in pLKO.1 puro vector (Engelman *et al*, 2007) and empty pLKO.1 puro vector were used for lentivirus production and infection in H441 cells as described (Engelman *et al*, 2007). Stably infected cells were selected in 2 *μ*g ml^−1^ of puromycin (Promega, Madison, WI, USA).

## Results

### Activation/overexpression of Met and resistance to GW2974

Activation of multiple RTKs simultaneously can contribute to the malignant phenotype in NSCLC. Thus, we used a RTK antibody array that captures 42 receptors and was then probed with antiphosphotyrosine to survey the activity of RTKs in NSCLC lines H441 and H1666. These lines have wild-type (WT) EGFR. Epidermal growth factor receptor and Her3 were highly activated in H1666 cells, and EGFR, Her2 and Her3 in H441 cells (Figure 1A). Both cell lines showed weak activation of MSPR and DTK, and some Her4 activity was detected in H441 cells (Figure 1A). In addition, H441 cells showed strong activation of Met, consistent with earlier reports (Christensen *et al*, 2003), whereas activated Met was not detected in H1666 cells (Figure 1A and C).

With the potential importance of ErbB receptor inhibitors for lung cancer treatment, and with multiple ErbB receptors activated in these cell lines, we investigated their sensitivity to the dual EGFR/Her2 kinase inhibitor GW2974 in proliferation assays. GW2974 is an analogue of lapatinib with an enzymatic acitivity of 0.007 *μ*M (compared with 0.012 *μ*M for lapatinib) for EGFR and 0.016 *μ*M (compared with 0.010 *μ*M for lapatinib) for Her2 (Rusnak *et al*, 2001; Petrov *et al*, 2006). Cell growth was assessed after a 5-day continuous exposure to drug. H1666 cells showed high sensitivity to GW2974 with an average GI_50_ of 0.1 *μ*M, compared with H441, where the GI_50_ was in the micromolar range (6–9 *μ*M; Figure 1B).

We examined the effects of GW2974 on signalling pathways both at the receptor level and for downstream signalling components (Figure 1C). GW2974 inhibited EGFR phosphorylation in H441 cells in a dose-dependent manner, although at micromolar concentrations. Surprisingly, phospho-Met was also inhibited by this compound at similar concentrations. Among potential downstream signalling components, phospho-Erk1/2 was not affected; however phospho-Akt, phospho-Stat3 and phospho-Shc were inhibited by GW2974 in a dose-dependent manner (Figure 1C). These data suggest that GW2974 affects Met activity, directly or indirectly, in H441 cells. In contrast, in H1666 cells, GW2974 inhibited phosphorylation of EGFR at submicromolar concentrations, as well as downstream signalling molecules including Akt, Erk1/2, and Stat3, consistent with its activity in the proliferation assay. Interestingly, there was little basal phosphorylation of EGFR at Y1068, or basal phosphorylation of Met at Y1234/1235 in H1666 cells, and the baseline phosphorylation of these receptors and downstream signalling molecules were only modestly inhibited by the drug (Figure 1C).

### Constitutive activation of Met, Her2 and Her3 in H441 cells is inhibited by GW2974

As NSCLC cells express many RTKs that could potentially contribute to GW2974 sensitivity, we used RTK antibody capture arrays to assess the effects of the drug on activation of 42 different receptors in parallel (Figure 2A). Cells were serum starved and then incubated with EGF for 10 min in the presence or absence of GW2974. Interestingly, Met, EGFR, Her2, Her3 and Ret were basally activated in H441 cells, even under serum-deprivation conditions. Stimulation with EGF further activated EGFR and Ret, but not Met, Her2 or Her3 (Figure 2A). The activation of all these receptors was inhibited by treatment with GW2974 (Figure 2A). A lighter exposure of RTK array experiment is shown as Supplementary Figure S1. Hence, EGFR, Met, Her2 and Her3 are all activated in this cell line without exogenous EGF or HGF. These data indicate constitutive activation of Met, Her2 and Her3 in this cell line, and cross-talk among the ErbB family receptors and Met.

Immunoblot analysis of these extracts confirmed the RTK array data showing constitutive activation of EGFR and Met and inhibition by GW2974 (Figure 2B and C). Epidermal growth factor was able to further activate the tyrosine phosphorylation of ErbB receptors, and this effect is abrogated by the 2 h treatment with GW2974 (Bands around 175 kDa correspond to ErbB receptors in Figure 2B). Detailed investigation of the effect of EGF treatment showed further phosphorylation of EGFR at Y845, but no further activation at Y1068 of EGFR and Met were observed (Figure 2C). Similarly, no further activation of Akt and Stat3 were observed with EGF treatment, whereas Shc and Erk1/2 were further activated by EGF treatment. Treatment of serum-deprived cells with GW2974 for only 2 h before EGF activation was sufficient to inhibit phosphorylation of EGFR and Met similar to 5-day continuous treatment with GW2974 in 10% serum (Figure 1C). GW2974 also reduced downstream phosphorylation of Stat3, Shc and Akt, although only reducing the EGF-dependent phosphorylation of Erk1/2 to baseline basal levels. Overall, these data show that GW2974 inhibits the activation of downstream signalling induced by EGF treatment.

### GW2974 does not inhibit HGF-dependent activation of Met in the absence of ErbB family receptors

The strong activation of members of both Met and ErbB families in H441 cells suggests two possible mechanisms for the inhibitory activity of GW2974 towards Met. The compound could work directly through an off-target inhibition of Met in these cells. Alternatively, ErbB receptors targeted by GW2974 may normally promote phosphorylation of Met in these cells. This would be consistent with recent studies suggesting receptor-level cross-talk between ErbB receptors and Met (Radaeva *et al*, 1999; Jo *et al*, 2000; Engelman *et al*, 2007).

**Figure 3:**
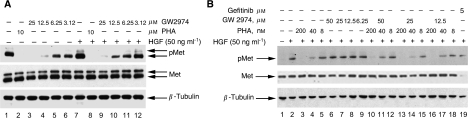
Cross-talk between Met and EGFR family receptors. GW2974 inhibits Met activation in a dose-dependent manner in H441 cells (**A**), but not in the absence of EGFR family receptors in 32D/Met cells (**B**). (**A**) GW2974 inhibits Met activation in the presence or absence of HGF. Cells were serum starved for 24 h followed by treatment with either vehicle (DMSO) or GW2974 or a Met inhibitor (PHA) for 2 h before activation with HGF (50 ng ml^−1^) for 30 min. Whole cell extracts were made and subjected to western blot with indicated antibodies. (**B**) GW2974 and gefitinib do not inhibit HGF-dependent activation of Met in stably transfected 32D cells with Met (32D/Met). Cells were treated as in panel A. *β*-Tubulin was used as a loading control.

To determine whether GW2974 directly inhibits the activation of Met, independent of its activity on EGFR and Her2, we incubated serum-starved H441 cells with the Met ligand, HGF. GW2974 inhibited phosphorylation of Met in the absence or presence of exogenous HGF in a dose-dependent manner similar to its activity in the presence of 10% foetal bovine serum (FBS) or EGF (Compare Figure 3A with Figures 1 and 2). In addition, Met is constitutively active in H441 cells (Figures 1 and 2), and we did not observe any further phosphorylation in the presence of HGF (Figure 3A). Furthermore, the activity of GW2974 on Met phosphorylation was not influenced by the presence of HGF in these cells. A small molecule inhibitor of Met (PHA665752, referred to hereafter as PHA) was used as a positive control for Met inhibition.

To directly evaluate possible off-target effects of GW2974 on Met, we assayed activity on myeloid 32D cells stably transfected with Met (32D/Met) (Day *et al*, 1999). These cells do not express endogenous ErbB family receptors (EGFR, Her2, Her3 and Her4). GW2974 did not strongly inhibit the HGF-dependent activation of Met (Figure 3B, lanes 6–9), although there may be a marginal reduction in Met activation at the higher concentration of GW2974. Moreover, combination of the Met inhibitor, PHA, with GW2974 yielded similar activity to PHA alone, and further reduction in phosphorylation of Met was not observed (Figure 3B, lanes 10–18). Another small molecule EGFR inhibitor, gefitinib, did not inhibit Met activation in 32D/Met cells (Figure 3B, lane 19 and Supplementary Figure S2). These data suggest that, in H441 cells, the activity of GW2974 is due to the inhibition of EGFR and/or Her2, which in turn affects the phosphorylation of Met. This suggests an active cross-talk between ErbB family receptors and Met.

### Physical interaction of Her2 and Met is inhibited by GW2974

**Figure 4:**
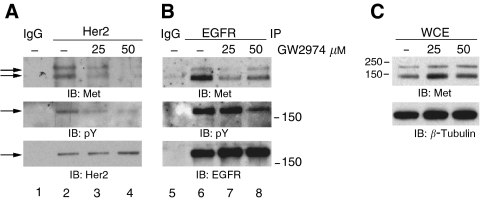
Met interacts with both EGFR and Her2, and the interaction is inhibited by GW2974. (**A** and **B**) Whole cell extracts were made from H441 cells treated with GW2974 for 2 h or DMSO vehicle control. Extracts (1 mg) were subjected to immunoprecipitation with anti-Her2 (**A**) or with anti-EGFR (**B**) antibodies followed by immunoblotting with indicated antibodies. Immunoprecipitation with IgG was used as a negative control in each experiment. Numbers on the side of panels indicate molecular weight marker. The middle panels of A and B are from the ErbB receptor region of the blot. (**C**) Whole cell extracts were immunoblotted with anti-Met antibody to indicate equal amounts of Met in each treatment. *β*-Tubulin was used as a loading control.

As GW2974 had little impact on Met in cells lacking ErbB family receptors, it is possible that, instead, the drug affects Met activation in H441 cells by inhibiting EGFR and/or Her2, and interfering secondarily with ErbB-dependent activation of Met. One possibility is that Met forms a physical complex with one or more ErbB family receptors. Indeed, Met coimmunoprecipitated with Her2, indicating that they are found in a protein complex (Figure 4A). This interaction was inhibited by GW2974 in a dose-dependent fashion (compare lanes 3–4 with lane 2) paralleling reduction in Met phosphorylation (Figure 1C). Similarly, Met was coimmunoprecipitated with EGFR in H441 cells (Figure 4B). However, in this case, GW2974 reduced, but did not completely abrogate the interaction (compare lanes 7–8 with lanes 3–4). These data suggest that the cross-talk of EGFR and Her2, with Met is mediated through interactions in a protein complex, although the nature of the interactions with EGFR and Her2 may be different.

### Downregulation of Met affects the activation of ErbB family receptors

**Figure 5:**
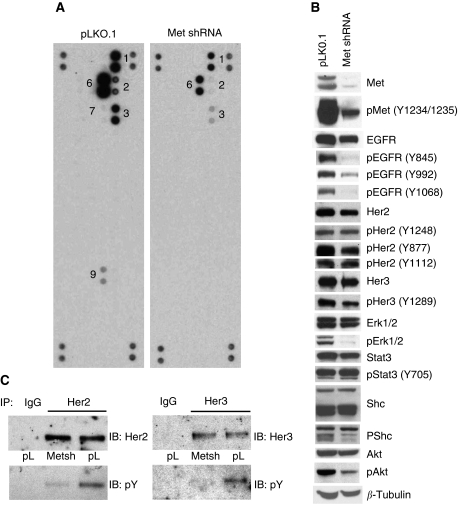
Met shRNA downregulates ErbB family receptor activation. Whole cell extracts were made from control (H441-pLKO.1) and Met shRNA (H441-Met shRNA) cells. (**A**) Whole cell extracts (200 *μ*g) were analysed on each RTK array membrane, and activation status of receptors was assessed using antiphosphotyrosine antibody and numbered as in Figure 1A. 1: EGFR; 2: Her2; 3: Her3; 6: Met; 7: MSRP; and 9: Ret. (**B**) The cell extracts analysed in panel A were analysed by immunoblotting with indicated antibodies. *β*-Tubulin was used as a loading control. (**C**) Cell extracts (500 *μ*g) used in panel A were subjected to immunoprecipitation using either anti-Her2 or anti-Her3 antibodies followed by immunoblotting with indicated antibodies. IgG was used as a negative control.

It has been shown previously that the downregulation of Met, in a Met-overexpressing cell line with activating mutation in EGFR, did not reduce the activation of EGFR or Her3, but did restore gefitinib sensitivity (Engelman *et al*, 2007). We examined the effect of Met downregulation using Met-specific shRNA in H441 cells, which strongly reduced the expression of Met, but not EGFR, Her2 or Her3 (Figure 5B). In RTK capture array analysis, not only the Met phosphorylation but also the phosphorylation of ErbB family receptors (EGFR, Her2 and Her3) and Ret (Figure 5A) were reduced. Total tyrosine phosphorylation of immunoprecipitated Her2 and Her3 was reduced with the expression of Met shRNA (Figure 5C), consistent with the RTK capture array data (Figure 5A). Further analysis of the effects of Met shRNA on ErbB family receptors revealed that tyrosine phosphorylation sites in ErbB receptors are affected differentially. Both TK domain (Y847) as well as autophosphorylation sites (Y992, Y1068) of EGFR are affected more, with concurrent inhibition of Erk1/2 and Akt phosphorylation. In comparison, autophosphorylation sites of Her2 (Y1289, Y1112) and kinase domain (Y877) site were only modestly affected along with only marginal effect on Shc and no apparent effect on Stat3 phosphorylation status. Similarly, Her3 at Y1289 was less affected by the downregulation of Met (Figure 5B). These data provide further evidence for the cross-talk of EGFR, Her2 and Her3 with Met.

### GW2974 inhibits wound healing in H441 cells

As Met interacts with both EGFR and Her2, and Met-dependent signalling is inhibited by GW2974, we investigated the effects of GW2974 on a process associated with Met biological activity, wound healing. Earlier study showed that wound healing in H441 cells is inhibited by treatment with the Met inhibitor PHA (Christensen *et al*, 2003). GW2974 inhibited scratch wound healing of H441 cells at a concentration that inhibits other Met-dependent signalling processes (Supplementary Figure S3). The scratch wound in cells treated with GW2974 (25 *μ*M) was not healed by 54 h (Supplementary Figure S3H), whereas the scratch wound in untreated cells healed by 24 h in the presence of 10% FBS (Supplementary Figure S3C). Similarly, the scratch wound of cells expressing Met shRNA did not heal by 24 h in the presence of 10% FBS, whereas the scratch-wounded control cells were healed by 24 h (Supplementary Figure S4B and D ).

### Combination of inhibitors of Met with ErbB family inhibitors

**Figure 6:**
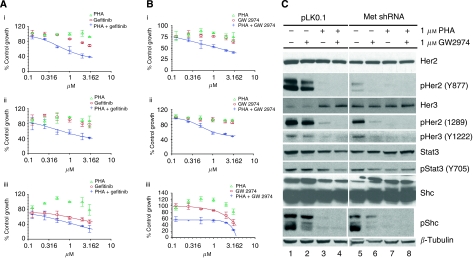
Synergistic inhibition of cell proliferation by combination of a Met inhibitor (PHA) with either gefitinib or GW2974 in H441 cells. Cells were treated with indicated concentrations of gefitinib and PHA (**Ai**–**iii**) or GW2974 and PHA (**Bi**–**iii**) either alone or in combination at 0.125–3 *μ*M concentration range for 5 days. For combination of drugs, a fixed ratio of 1 was used. Cell proliferation was assessed as in Figure 1B. Data were plotted as dose–response regression curves using Xlfit program. Each error bar represents the s.d. from three data sets. **Ai**–**Bi**: H441 cells; **Aii**–**Bii**: H441-pLKO.1 cells; and **Aiii**–**Biii**: H441-Met shRNA cells. (**C**) Whole cell extracts were made from cells treated with drugs for 5 days as indicated, and were subjected to western blot analysis with indicated antibodies. *β*-tubulin was used as a loading control.

To explore the therapeutic implications of Met activation in the treatment of NSCLC, we combined the Met inhibitor with either gefitinib (Figure 6A) or GW2974 (Figure 6B) and monitored cell growth in H441 cells, H441-pLKO.1 and H441-Met shRNA cells. A fixed ratio of 1 was used for both PHA and GW2974 with concentrations ranging from 0.125 to 3 *μ*M. Each data set is plotted as a dose–response regression curve. Each drug alone was unable to effectively inhibit the proliferation at concentrations used in this study, but the combination of two drugs resulted in a synergistic inhibition of proliferation. Importantly, the downregulation of Met restored both gefitinib and GW2974 activities (Figure 6Aiii and Biii). Western blot analysis indicates that GW2974 is more effective in inhibiting phosphorylation of Her2, Her3, Stat3 and Shc in H441-Met shRNA cells compared with control-infected H441-pLKO.1 cells (Figure 6C) suggesting that Met is responsible for the activation of EGF family receptor-dependent signalling and resistance to GW2974 in H441 cells.

The combination of GW2974 with PHA yielded greater synergistic inhibition at lower concentration of both drugs than gefitinib with PHA for cell growth in H441-Met shRNA cells (compare Figure 6Aiii with Figure 6Biii), suggesting that Met directly affects the activation of EGFR in H441 cells. These data, when combined with other data presented in this study, suggest that simultaneous inhibition of multiple RTKs may be needed to effectively abrogate cell growth.

## Discussion

In an effort to better understand potential druggable targets in NSCLC, we screened several cell lines with RTK capture arrays. These experiments revealed that, in H441 cells, the dominant RTK activity was for EGFR, Her2, Her3 and Met receptors. The H441 cell line has been previously shown to be resistant to gefitinib and cetuximab (Tracy *et al*, 2004; Mukohara *et al*, 2005). We found that the activity of EGFR in these cells can be inhibited by the dual EGFR and Her2 kinase inhibitor GW2974 at high micromolar concentrations, which also affects the phosphorylation of Stat3, Akt and Shc, but not Erk1/2. Unexpectedly, this inhibitor also reduced the high level of basal Met phosphorylation. The inhibition of Met by GW2974 seems to occur mainly by an indirect mechanism, as this compound has no effect on Met phosphorylation in Met-expressing 32D cells (32D/Met) that lack EGFR and Her2 receptors. As Met is coimmunoprecipitated with Her2 and EGFR, and this interaction is inhibited by GW2974, it is suggested that the disruption of physical complexes among RTKs is one possible mechanism. GW974 does have strong biological activities that probably work through Met, including the inhibition of wound healing after scratch injury of a cell monolayer. Moreover, combinations of anti-Met and anti-ErbB drugs yielded synergistic effects on cell proliferation and on downstream signalling from these receptors.

Activated EGFR, Her2, Her3 and Her4 form heterodimers that lead to cooperative and synergistic outputs. The close cooperativity of EGFR with Her2 has led to the development of other ErbB receptor family inhibitor drugs including the dual EGFR/HER2 TKI lapatinib and therapeutic antibody, pertuzumab, which blocks Her2 interactions with EGFR and Her3. These drugs are currently in clinical trials (pertuzumab) or approved (lapatinib), showing their promising activity in cancer. These receptors are also modulated by direct association with other receptors (including gp130), and by the impact of other receptors on functional activation of growth factor propeptides by proteolytic processing (e.g., Hb-EGF activation by G-protein-coupled receptors).

To date, there are no targeted therapies that are appropriate for the majority of NSCLC patients who do not respond to erlotinib or gefitinib. Presumably, these cancers are driven by the activation of other signalling molecules beyond the EGFR. Some will have mutations in other RTKs that would be hallmarks of sensitivity to RTK inhibitors with the appropriate action spectrum, whereas others may be driven by mechanisms unrelated to RTKs. Profiling tumours with RTK capture arrays may be very helpful in first-pass analysis of tumours to identify active RTKs that may be susceptible to treatment with the kinase inhibitors. For example, our finding that H441 cells are sensitive to Met, and ErbB inhibitors could be predicted based on the activity of these receptors determined by RTK capture array (Figure 1).

Besides posing treatment challenges, the cross-interactivity of RTKs potentially provides new treatment opportunities. This study is focused on the interactions between two RTK families, ErbBs and Met, that have overlapping functions in initiation and progression of cancer. ErbBs are implicated in promoting proliferation and survival, and Met has important roles in cell motility and epithelial/mesenchymal transition. Our identification of ErbB and Met signalling in a single lung cancer cell line, and the involvement in responsiveness to ErbB and Met inhibitors is consistent with other recent studies that highlighted functional cooperation between these two receptor families (Bean *et al*, 2007; Guo *et al*, 2008; Mueller *et al*, 2008; Shattuck *et al*, 2008; Yano *et al*, 2008). Interaction of these receptor families has been suggested for years, based on the finding that Met is often co-overexpressed with ErbBs in prostate and breast carcinoma. Hepatocyte growth factor promotes motility and invasive behaviour by Her2 (Neu)-transformed mammary epithelial cells (Khoury *et al*, 2005). A recent phosphoproteomic screen for EGFR targets in glioblastoma identified Met (Stommel *et al*, 2007).

Receptor tyrosine kinase antibody arrays used in this study clearly indicated differences in receptor activation levels in two cell lines with WT EGFR, which prompted us to investigate ErbB family receptor inhibitors in NSCLC. In this study, we report the activity of a lapatinib analogue, GW2974, which is a small molecule dual TKI of EGFR/Her2. Lapatinib has shown preferential benefit for Her2 amplified breast cancer patients compared with patients with normal level of Her2 (Konecny *et al*, 2006). Although HER2 is not commonly amplified in NSCLC, in approximately 2% of NSCLC patients, an activating mutation similar to those observed in EGFR has been reported in Her2. The response rate for lapatinib alone in a randomised clinical trial for NSCLC was very low (Smylie *et al*, 2007). We have shown that GW2974 does show a high micromolar activity in H441 cells that carry WT EGFR and constitutive activation and expression of Met. This activity is similar to non-amplified Her2 in breast cancer (Konecny *et al*, 2006). However, we report a GW2974-sensitive lung cancer cell line (H1666) that has normal level of both Her2 and Met, and does not have activating mutation in EGFR. GW2974 was able to downregulate EGFR-dependent activation of Akt, Erk1/2, and had a marginal effect on Stat3 in a dose-dependent fashion (Figure 1C). This cell line has been shown to be resistant to gefitinib. Sensitivity of this cell line to GW2974 is similar to its activity in Her2 amplified breast cancers. Further investigation is necessary to understand the molecular mechanism for its response to this drug.

On the contrary, GW2974 downregulated Met-dependent signalling in H441 and showed no effect on the phosphorylation level of Erk1/2. Constitutive activation of Met, Her2 and Her3 even in serum-starved cells suggests a ligand-independent activation of these receptors. One possibility may be autocrine activation of these receptors due to the expression of a ligand, such as EGF or HGF etc. The expression of ligand by cancer cells has been observed in many cases that often lead to drug resistance bypassing a need for the overexpression or activating mutation in oncogenes for cancer progression. A second possibility is the transactivation by another receptor, for example, Met in H441 cells that bypasses the need for ligand-dependent activation of EGFR, Her2 and Her3 (Figures 1, 2, 5 and 6). GW2974 inhibited the proliferation, Met-dependent activation of downstream signalling and phosphorylation of Met in a dose-dependent manner in H441 cells, but did not downregulate the HGF-dependent activation of Met in 32D/Met cells suggesting that in H441, there is an active cross-talk between Met and ErbB receptors. However, the inhibitory activity of GW2974 is observed at higher (micromolar) concentration than is required normally for the inhibition of EGFR/Her2-dependent signalling. In H441 cells, GW2974 only inhibited the activation of EGFR, Her2 and Her3 at lower concentrations, when Met was downregulated (Figure 6, compare lane 2 with lane 6). Indeed, both Her2 and EGFR are able to coimmunoprecipitate Met in H441 cells. The interaction of Met with Her2 is inhibited by GW2974 in a dose-dependent fashion similar to its activity towards Met-dependent signalling. However, the interaction of Met with EGFR is of a different nature, as this interaction is inhibited by GW2974 without showing any dose dependence. For the first time, we have shown that the downregulation of Met by Met-specific shRNA leads to differential effect on the tyrosine phosphorylation sites of EGFR, Her2 and Her3 (Figure 5) suggesting that Met plays a crucial role in activating ErbB family receptors especially EGFR, as the activation of EGFR is drastically affected with concomitant inhibition of Erk1/2 and Akt activation. However, activation of Stat3 was not affected, and Shc is only marginally affected by Met downregulation suggesting that it is regulated by different mechanism. One such mechanism could be ligand-dependent activation of Her2 and Her3 in these cells, as the activation of both are downregulated by GW2974 in a dose-dependent manner.

To explore the therapeutic implications of coactivation of RTKs, we evaluated the combination of inhibitors for Met and ErbB family receptors in H441 cells. As shown in Figure 6, either drug alone was not very effective at inhibiting proliferation. However, the combination of drugs was synergistic in inhibiting the proliferation of cells very effectively. Interestingly, it is very clear from regression curves that any small inhibition by PHA alone was sufficient to effectively inhibit the cell growth when combined with GW2974 at the lowest concentration of 0.125 *μ*M in Met shRNA cells (Figure 6Biii), whereas this was not the case with PHA and gefitinib in the same cells (Figure 6Aiii). This suggests that the coactivation of multiple RTKs in cancer cells confer resistance to single-agent therapy, but this resistance can easily be overcome by simultaneously inhibiting several RTKs with rationally chosen drug combinations. Similar observations were made recently in the case of glioblastomas carrying activated EGFR and Met receptors where simultaneous inhibition of EGFR (erlotinib) and Met (SU11274) lead to the downregulation of PI3K pathway, and reduced the size of colony formation in soft agar (Stommel *et al*, 2007). The single inhibition by single drug was less effective. Thus, our study combined with other similar observations provides a compelling evidence to effectively treat cancers by rationally designed drug combinations.

The rapid pace of development of molecularly targeted anticancer drugs creates a concomitant need for the development of the best tools for matching the drugs to appropriate patients. Phosphoproteomic analysis by RTK capture arrays may be a valuable tool for identifying the subset of tumours with functional receptor activation, regardless of mechanism. Similar arrays that monitor the activity of intermediary signalling molecules through their phosphorylation status provide one relatively inexpensive tool for this purpose. The best analysis of tumour samples may require a combination of proteomic, genetic, epigenetic and functional characterisation.

The functional studies here led to the hypothesis that dual targeting of ErbBs and Met may be very important in the treatment of some human cancers. Ectopic signalling by members of each receptor family are common in human cancer, and the receptor systems have complementary functions in promoting growth, survival and qualities associated with invasion and metastasis. This study when combined with other studies provides compelling support for the combination of these drugs in future clinical trials.
