# Supplementary figures and images for: Association of constitutively activated hepatocyte growth factor receptor (Met) with resistance to a dual EGFR/Her2 inhibitor in non-small-cell lung cancer cells
Source: Br J Cancer. 2009 Feb 24;100(6):941–9. doi: 10.1038/sj.bjc.6604937 (PMC2661782; doi:10.1038/sj.bjc.6604937)

supplemental Figure S1, Agarwal et al

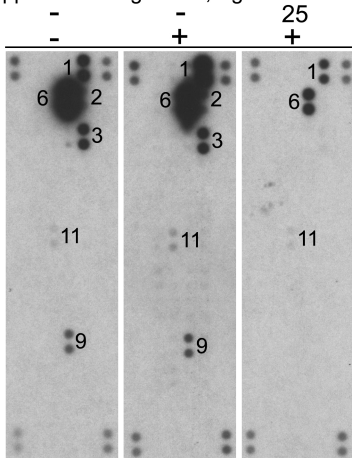

GW2974,  $\mu\text{M}$   
EGF, 100 ng/ml

Supplement: Supplementary Figure S1 [file 6604937x1.pdf]

Agarwal et al  
Supplemental Figure S2

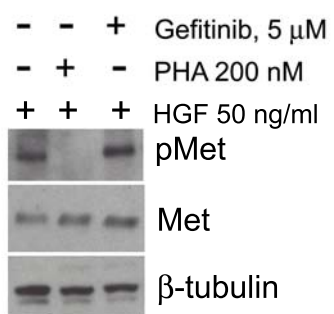

Supplement: Supplementary Figure S2 [file 6604937x2.pdf]

Supplemental Figure S3  
Agarwal et al

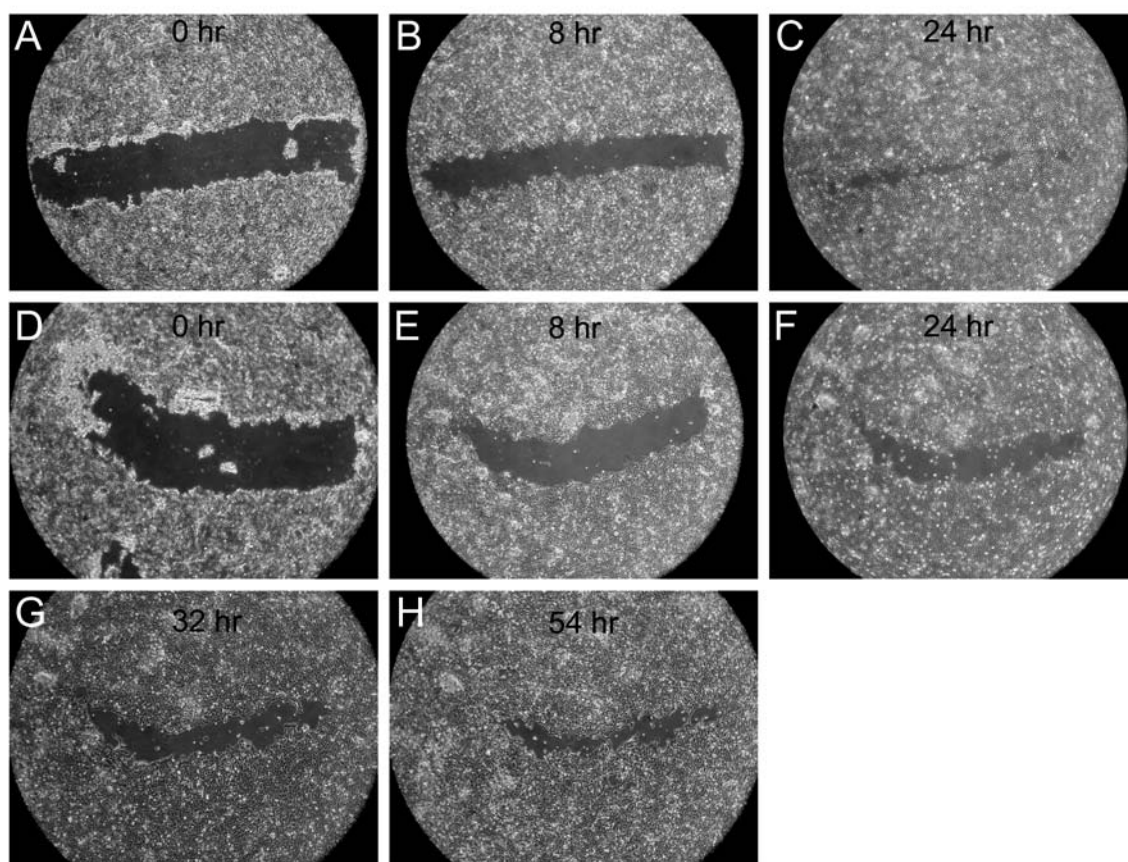

Supplement: Supplementary Figure S3 [file 6604937x3.pdf]

Supplemental Figure S4  
Agarwal et al

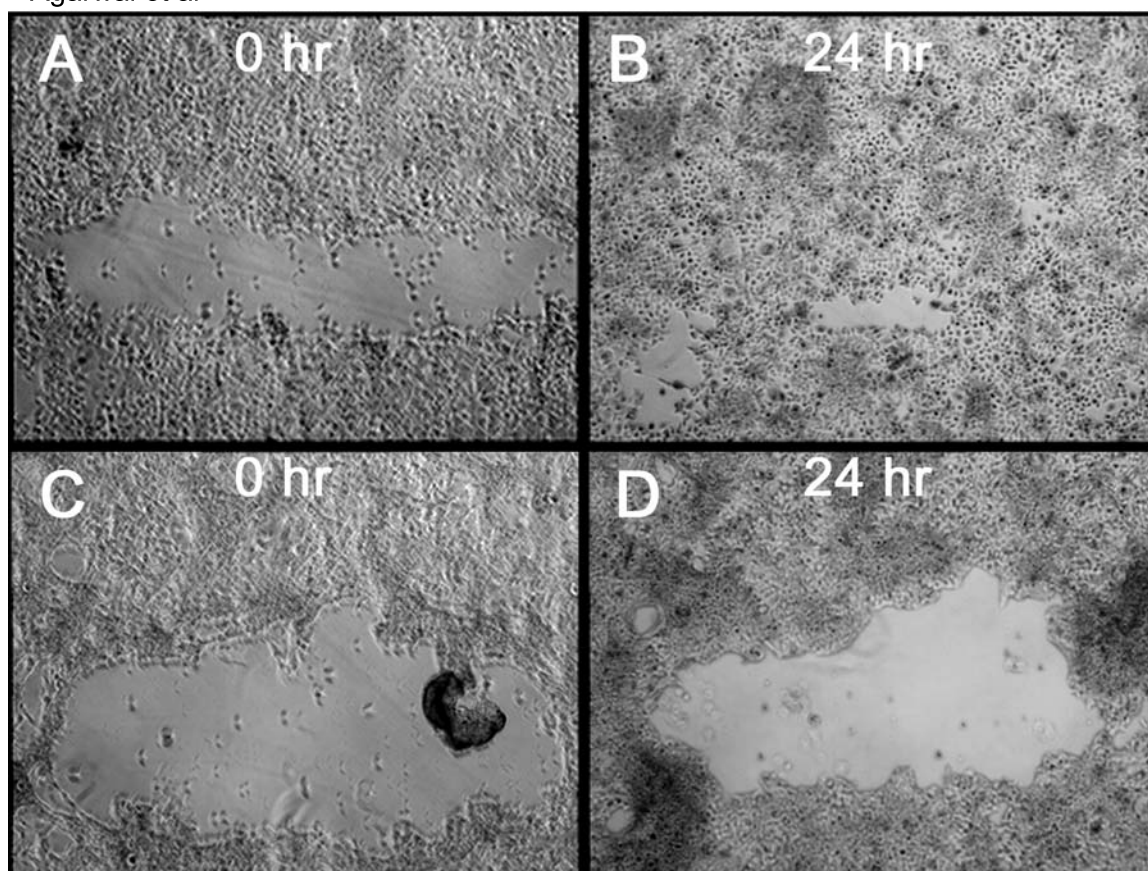

Supplement: Supplementary Figure S4 [file 6604937x4.pdf]
