# Supplementary material for: Association of constitutively activated hepatocyte growth factor receptor (Met) with resistance to a dual EGFR/Her2 inhibitor in non-small-cell lung cancer cells
Source: Br J Cancer. 2009 Feb 24;100(6):941–9. doi: 10.1038/sj.bjc.6604937 (PMC2661782; doi:10.1038/sj.bjc.6604937)
Supplement: Supplementary Material [file 6604937x5.pdf]

Supplemental Material

Material and Methods

RTK-antibody array profile: Whole cell extracts were made in lysis buffer containing 20 mM Tris-HCl (pH 8.0), 150 mM Nacl, 1% NP-40, 10% glycerol, 2 mM EDTA, 1 mM sodium orthovanadate, 1xprotease inhibitor cocktail (complete mini-EDTA from Roche scientific), and 1 mM DTT. Following 30 min incubation on ice, cells were scraped and cleared by centrifugation at 14000 rpm for 10 min at 4^0C. Protein concentrations were determined using Bio-Rad protein assay reagent. Either 200 µg (Fig 1A) or 500 µg (Fig 2A) were used as suggested by the manufacturer.

Immunoprecipitation and immunoblot: For the immunoprecipitation (IP) experiment, cells were incubated with either vehicle (0.5% DMSO) or with indicated concentrations of GW2974 in 0.5% DMSO containing cell media for 2 hr. Cells were immediately washed in cold PBS. Whole cell extracts were made as in RTK-antibody array profile. EGFR and Her2 complex were immunoprecipitated using mouse anti-EGFR or mouse anti-Her2 antibody with total of 1 mg of protein. Protein A sepharose beads (Amersham Bioscience) were used to capture the complex and were washed in IP buffer and eluted by boiling in 1x SDS-PAGE loading buffer for 5 min. 50% of eluted proteins from each IPs were used to blot with antibody against Met. Another 25% of eluted protein was used to perform WB using anti-phospho-tyrosine antibody and the remaining 25% was used to determine the total EGFR (for EGFR IP) or Her2 (for Her2 IP) protein level. Proteins were separated in NuPage 4-12% gradient Bis-Tris gels, transferred to nitrocellulose, blocked in 5% non-fat dry milk in TBST and blotted with antibodies as indicated. Proteins were detected using secondary antibody either goat anti-mouse-HRP or goat anti-rabbit-HRP. Whole cell

extracts were also separated, transferred and detected for total and phosphorylated proteins using appropriate antibodies.

HGF activation: Cells were plated in 24-well plate in 750 µl of starvation media (no FBS and no IL-3 containing WEHI media) at a density of $5x10^6$ cells/well for 32D/Met cells. After 2 hr of starvation, compounds were serially diluted into cell starvation media as 2× stocks containing 1% DMSO and 750 µl of each dilution was added to the wells after 2 hr of starvation. Additional 2 hr incubation with compound in starvation media, cells were activated with 50 ng/ml of HGF for 5 min. Cells were collected immediately in 2 ml eppendorf tubes and spun down for 2 min at 4^0C, washed once with PBS followed by addition of 100 µl of lysis buffer in each tube. After 30 min of incubation at 4^0C the lysates were cleared by centrifugation at 14000 rpm for 10 min at 4^0C. Protein estimation was performed and equal amounts of proteins were separated on NuPage 4-12% gradient Bis-Tris gels as mentioned above. Total Met and phospho-Met levels were determined by WB using appropriate antibodies. Beta-tubulin was used as loading control.

Wound healing assay: For wounding assay, confluent cells were scraped using a sterile pipette tip, washed once with PBS to remove all floating dead cells. Immediately treated or left untreated (0.5% DMSO control) with appropriate concentration of compound in 10% FBS media containing 0.5% DMSO. Wound healing was monitored by photographing each scratch at different time intervals. Each treatment was done at least in triplicate and repeated several times.

Figure Legend.

Figure S1. Same RTK array as in Figure 2A, but a different exposure.

Figure S2. Gefitinib does not inhibit the phosphorylation of Met in 32D/Met cells. Western blot analysis of same samples as in Figure 3B lanes 2, 3 and 19.

Figure S3. GW2974 inhibits wound healing in H441 cells. A-H. Confluent layer of cells were wounded by scratch followed by treatment with DMSO (A-C) or with GW2974 (25 μM, D-H) and photographed at indicated times. Representative data are shown from results of multiple wounds.

Figure S4. Confluent layer of H441-pLKO.1 and H441-Met shRNA cells were wounded by scratch and photographed at indicated times. Representative data are shown from the results of multiple wounds. A-B. H441-pLKO.1; and C-D. H441-Met shRNA cells.
